# Challenges and Lessons Learned From a Mobile Health, Web-Based Human Papillomavirus Intervention for Female Korean American College Students: Feasibility Experimental Study

**DOI:** 10.2196/14111

**Published:** 2020-01-29

**Authors:** Minjin Kim, Haeok Lee, Jeroan Allison

**Affiliations:** 1 University of Massachusetts Medical School Department of Population and Quantitative Health Sciences Worcester, MA United States; 2 University of Massachusetts Boston College of Nursing and Health Sciences Boston, MA United States

**Keywords:** mHealth, Web-based intervention, fraud, experimental design

## Abstract

**Background:**

Mobile health (mHealth) and Web-based research methods are becoming more commonplace for researchers. However, there is a lack of mHealth and Web-based human papillomavirus (HPV) prevention experimental studies that discuss potential issues that may arise.

**Objective:**

This study aimed to assess the feasibility of research procedures and discuss the challenges and lessons learned from an mHealth and Web-based HPV prevention experimental study targeting female Korean American college students in the United States.

**Methods:**

A pilot randomized controlled trial (RCT) was conducted in an mHealth and Web-based platform with 104 female Korean American college students aged 18-26 years between September 2016 and December 2016. Participants were randomized to either the experimental group (a storytelling video intervention) or the comparison group (a nonnarrative, information-based intervention). Outcomes included the feasibility of research procedures (recruitment, eligibility, randomization, and retention).

**Results:**

From September 2016 to October 2016, we recorded 225 entries in our initial eligibility survey. The eligibility rate was 54.2% (122/225). This study demonstrated a high recruitment rate (95.6%, 111/122) and retention rate (83.7%, 87/104) at the 2-month follow-up.

**Conclusions:**

Findings from this study demonstrated sufficient feasibility in terms of research procedures to justify a full-scale RCT. Given the increased possibility of invalid or misrepresentative entries in mHealth and Web-based studies, strategies for detection and prevention are critical.

**Trial Registration:**

ISRCTN Registry ISRCTN12175285; http://www.isrctn.com/ISRCTN12175285

## Introduction

### Background

Korean American women (11.9 per 100,000) are disproportionately affected by cervical cancer compared with the overall population of women in the United States (7.2 per 100,000) [1]. Korean American women have been consistently identified as the least likely subgroup to receive cervical cancer screenings [2,3]. Despite growing evidence showing the benefits of human papillomavirus (HPV) vaccines in preventing cervical cancers, there are few population-based data on HPV vaccination behavior and intervention studies to promote HPV vaccination behavior for young Korean Americans [4,5].

Mobile health (mHealth) and Web-based research methods are increasingly common as a tool for research and research settings [6]. In particular, online outreach is the most effective recruitment method for young, bilingual Korean Americans [7]. Despite this significant potential, there are minimal HPV intervention studies that discuss issues specific to mHealth and Web-based studies of particular populations.

### Objective

To fill this gap, this study aimed to (1) assess the feasibility of research procedures (recruitment, eligibility, randomization, and retention) to inform a future randomized controlled trial (RCT) of an mHealth and Web-based HPV prevention program targeting young Korean Americans and (2) discuss the challenges and lessons learned from an mHealth and Web-based study.

## Methods

### Study Design

This was a pilot RCT that consisted of multiple stages: (1) a qualitative study [8] including an HPV storytelling intervention [9], (2) assessment of the acceptability of the intervention [9], (3) assessment of the feasibility of mHealth and Web-based research procedures, and (4) evaluation of the preliminary effectiveness of the intervention [4]. This study reports on the feasibility of research procedures in light of a 2-month follow-up.

**Figure 1:**
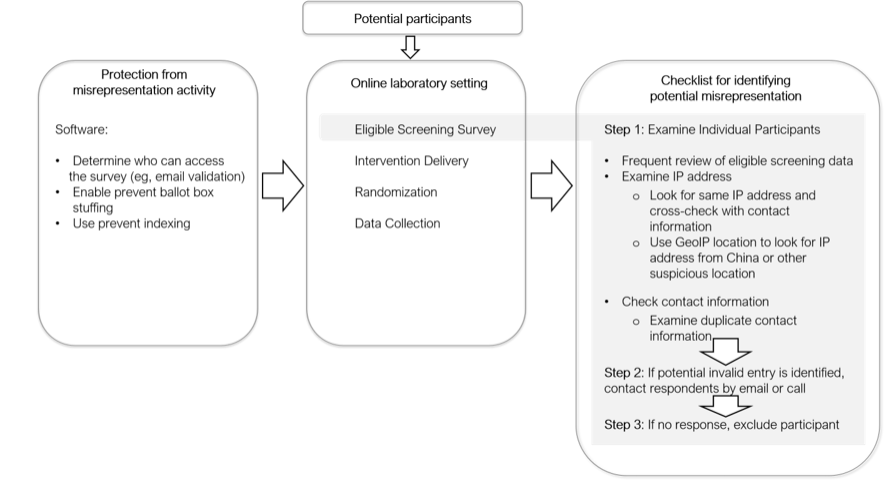
The process of preventing and detecting misrepresentation entries. IP: internet protocol.

The study protocol was designed to maintain data and research integrity (Figure 1). We used personally identifiable information (eg, name, email address, phone number, and internet protocol [IP] address) to detect multiple attempts or duplication in the online survey. The protocol was explained in the consent form provided. The institutional review board at the University of Massachusetts Boston approved the research procedures of this study.

### Recruitment Process

From September 2016 to October 2016, we recruited participants via social networking sites (eg, Facebook and KakaoTalk), Korean community websites, and word-of-mouth sources, including Korean church communities and Korean student associations in the northeast. Recruitment materials focused on the following theme: “I Want to Know More about the HPV Vaccine 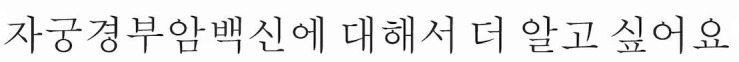.” Potential subjects were instructed to visit the study website.

### Eligibility Criteria

We anticipated that some respondents would be ineligible for the study. Therefore, we used a 4-question online survey to ensure that respondents were female Korean American college students (aged 18-26 years) from the northeastern United States who had not yet been vaccinated against HPV. Eligible participants received a link to our mHealth and Web-based study platform where we collected data, disseminated the intervention, performed randomization, and accommodated mobile devices and Web services.

### Participant Randomization

At the end of the baseline survey, participants were randomly assigned to either the experimental group (a storytelling video intervention) or the comparison group (a nonnarrative, written statement about HPV and HPV vaccine) using the randomizer tool in Qualtrics survey software that offers the mobile and Web-based platform, until each group contained 60 participants.

### Study Retention

Furthermore, 2 months after the intervention, participants received a follow-up survey via email with a passcode. In total, two reminders about the follow-up survey were sent via email. Each individual received a US $20 electronic gift (e-gift) card at the end of the postintervention survey and was entered into a drawing for an e-gift card at the end of the follow-up survey.

### Feasibility Outcome

The feasibility and validity of mHealth and Web-based research procedures were measured as follows: (1) recruitment rate was measured for the percentage of participants who signed the informed consent forms out of the total number of eligible participants, (2) eligibility rate was measured by dividing the number of total entries in the eligibility screening survey by the number of individuals who met the inclusion criteria, (3) randomization was measured by whether we were able to randomize eligible participants evenly, and (4) retention rate was measured by the 2-month follow-up participation. In addition, we identified the recruitment source by asking how participants had heard of the study.

## Results

### Participant Selection

**Figure 2:**
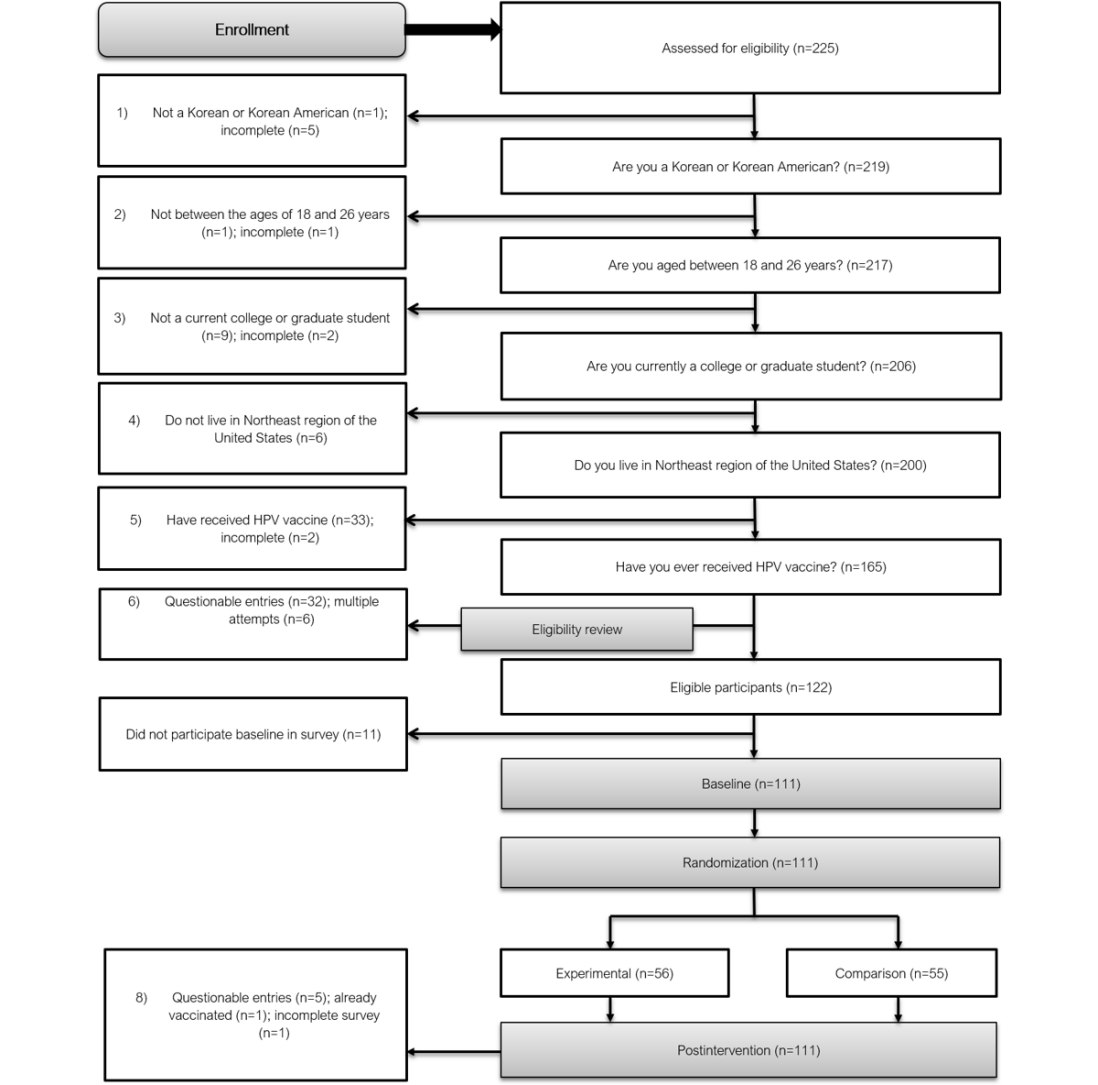
Flow chart. HPV: human papillomavirus.

Figure 2 shows the flow of participants throughout the pilot RCT.

### Recruitment Rate

Between September 2016 and October 2016, the recruitment target of 60 participants in each group was achieved. Out of 225 potentially eligible participants, 204 individuals responded to how they heard about the study. The most commonly reported recruitment methods were through friends 46.1% (94/204) and social media 33.8% (68/204). The recruitment rate was 95.6% (111/122).

### Eligibility Rate

The eligibility rate was 54.2% (122/225) after excluding ineligible participants (n=60), multiple attempts at entries (n=6), and questionable entries (n=37; Figure 2).

### Randomization

Randomization between groups was effectively demonstrated through the Qualtrics software. However, after ineligible and incomplete cases were excluded, an uneven number of participants remained in each group: 56 in the experimental group and 55 in the comparison group.

### Retention Rate

In total, 83.7% (87/104) of participants participated in the 2-month follow-up study: 45 in the intervention group and 42 in the comparison group.

## Discussion

### Principal Findings

This study assessed the feasibility of using an mHealth and Web-based HPV intervention experimental study with young Korean Americans to inform a future RCT. Overall, these findings demonstrate that the mHealth and Web-based experimental study is feasible if software protection and a systemic approach to eligibility screening are used. This study demonstrated a high recruitment rate (95.6%, 111/122) and a moderate eligibility rate (54.2%, 122/225). Although our eligibility rate was moderate because of restrictive inclusion criteria, we believe that our recruitment strategies filled the gaps of previous studies’ concerns in recruiting Korean Americans [10,11] by achieving the target sample size in 2 months of recruitment activities. Our approach suggests a way to overcome this challenge. Randomization was evenly distributed between the groups, although ineligible and incomplete cases led to an uneven number of participants in each group in the dataset. In this study, the retention rate was high (83.7%, 87/104) at the 2-month follow-up. In future RCT research, encouraging reminders are recommended as a method to improve HPV vaccine participation and compliance and increase engagement, minimize attrition, and maximize retention.

### Challenges and Lessons Learned From the Data Validation Process

Several challenges in the process provide important lessons for future mHealth and Web-based experimental studies. One challenge was the prevention and detection of multiple attempts at entries and potential misrepresentation, which could threaten the validity of the study. We addressed this challenge with software protection, such as *Prevent Indexing* and *Prevent Ballot Box Stuffing* (which prevents participants from taking a survey multiple times by using browser cookies), as well as a systemic approach for eligibility screening to prevent and detect multiple attempts at entries and identify potential misrepresentation.

Despite these precautions, we found a considerable number of invalid entries. These entries had similar or identical IP addresses, and contact information and IP addresses were from outside the northeastern United States. These activities may be explained by our use of monetary incentives, which can not only enhance recruitment and retention in mHealth and Web-based studies [12] but also encourage ineligible applicants and multiple attempts by eligible individuals. Studies using electronic health have discussed similar challenges [13,14] and suggested that a systematic approach for eligibility screening may help obtain valid data [13-15]. In addition, we believe it is critical to cross-check the respondent’s information (eg, email address, phone number, and demographic information) with the information collected during the eligibility screening and after the data collection. We found 6 cases of multiple attempts during the eligibility screening and additional 5 cases of multiple attempts via the screening of IP addresses and contact information after the postintervention survey when we reviewed their contact information to send incentives.

Ultimately, it may be necessary to use an email validation feature to ensure that respondents are entering a valid email address. For example, researchers can set a condition to include *edu* in the first email field and a logic condition to require that verification email to be equal to the first email. In this way, researchers may decrease invalid or multiple entries and confirm the verification of respondents’ student status regardless of recruitment methods. Moreover, researchers can block questionable IP addresses or contact information using a survey software.

To protect the integrity of mHealth and Web-based research data, researchers and institutional review boards must be mindful about possible misrepresentation activities and how or what to take action on when these activities are suspected or detected. In future research studies, we suggest researchers develop a data and safety monitoring plan regarding how to maintain research integrity if an invalid activity is suspected or detected.

To our knowledge, this is the first study to examine the feasibility of an mHealth and Web-based experimental study to promote HPV vaccination among young Korean Americans who are at a high risk for HPV infections and HPV-attributable cancers. These findings may not be generalizable to other populations.

### Conclusions

An mHealth and Web-based HPV prevention experimental study is an efficient and flexible way to perform research with female Korean American college students. However, given the increased possibility of invalid and multiple entries in mHealth and Web-based studies, strategies for detection and prevention are critical. Findings from this study demonstrated sufficient feasibility in terms of research procedures—including recruitment, data collection, randomization, and retention—to justify a full-scale RCT. The lessons learned from this study provide key insights into other future mHealth and Web-based experimental studies.

## Appendix

Multimedia Appendix 1 CONSORT‐EHEALTH checklist (V 1.6.1).

## Abbreviations

e-gift: electronic gift
HPV: human papillomavirus
IP: internet protocol
mHealth: mobile health
RCT: randomized controlled trial

## References

[1] Wang SS, Carreon JD, Gomez SL, Devesa SS (2010). Cervical cancer incidence among 6 asian ethnic groups in the United States, 1996 through 2004. Cancer.

[2] Han HR, Song Y, Kim M, Hedlin HK, Kim K, Ben Lee HB, Roter D (2017). Breast and cervical cancer screening literacy among Korean American women: a community health worker-led intervention. Am J Public Health.

[3] Fang CY, Ma GX, Handorf EA, Feng Z, Tan Y, Rhee J, Miller SM, Kim C, Koh HS (2017). Addressing multilevel barriers to cervical cancer screening in Korean American women: a randomized trial of a community-based intervention. Cancer.

[4] Kim M (2017). ScholarWorks at UMass Boston.

[5] Kim M, Lee H, Kiang P, Aronowitz T, Sheldon LK, Shi L, Kim S, Allison J (2019). HPV vaccination and Korean American college women: cultural factors, knowledge, and attitudes in cervical cancer prevention. J Community Health.

[6] Francis DB, Cates JR, Wagner KP, Zola T, Fitter JE, Coyne-Beasley T (2017). Communication technologies to improve HPV vaccination initiation and completion: a systematic review. Patient Educ Couns.

[7] Park H, Sha M (2015). Do different recruitment methods reach different Asian demographics?. Surv Pract.

[8] Kim M, Lee H, Kiang P, Kim D (2017). Human Papillomavirus: a qualitative study of Korean American female college students' attitudes toward vaccination. Clin J Oncol Nurs.

[9] Kim M, Lee H, Kiang P, Allison J (2019). Development and acceptability of a peer-paired, cross-cultural and cross-generational storytelling HPV intervention for Korean American college women. Health Educ Res.

[10] Katigbak C, Foley M, Robert L, Hutchinson MK (2016). Experiences and lessons learned in using community-based participatory research to recruit Asian American immigrant research participants. J Nurs Scholarsh.

[11] Im EO, Lee Y, Ji X, Zhang J, Kim S, Chee E, Chee W, Tsai H, Nishigaki M, Yeo SA, Shapira MM, Mao JJ (2016). Internet recruitment of Asian American breast cancer survivors. ANS Adv Nurs Sci.

[12] DeCamp W, Manierre MJ (2016). 'Money Will Solve the Problem': testing the effectiveness of conditional incentives for online surveys. Surv Pract.

[13] Kramer J, Rubin A, Coster W, Helmuth E, Hermos J, Rosenbloom D, Moed R, Dooley M, Kao Y, Liljenquist K, Brief D, Enggasser J, Keane T, Roy M, Lachowicz M (2014). Strategies to address participant misrepresentation for eligibility in web-based research. Int J Methods Psychiatr Res.

[14] Teitcher JE, Bockting WO, Bauermeister JA, Hoefer CJ, Miner MH, Klitzman RL (2015). Detecting, preventing, and responding to 'fraudsters' in internet research: ethics and tradeoffs. J Law Med Ethics.

[15] Quach S, Pereira JA, Russell ML, Wormsbecker AE, Ramsay H, Crowe L, Quan SD, Kwong J (2013). The good, bad, and ugly of online recruitment of parents for health-related focus groups: lessons learned. J Med Internet Res.

